# Supplementary material for: Assessing the Impact of Workforce Nutrition Programmes on Nutrition, Health and Business Outcomes: A Review of the Global Evidence and Future Research Agenda
Source: Int J Environ Res Public Health. 2023 May 5;20(9):5733. doi: 10.3390/ijerph20095733 (PMC10178561; doi:10.3390/ijerph20095733)
Supplement: Supplementary file 1 [file ijerph-20-05733-s001.zip › ijerph-2291398-supplementary.pdf]

Supplementary Material Document

Data charting form 1: Article details and general information

No.	Article Title	Author(s), Year	Study Type	Primary aim(s)	Country(ies)	Income level(s)[1]	Workforce nutrition pillar(s)[2]	Outcome measures[3]	Programme duration
**1**	Impact of a Worksite Diabetes Prevention Intervention on Diet Quality and Social Cognitive Influences of Health Behavior: A Randomized Controlled Trial	Miller et al. [17]	Randomized controlled trial (RCT)	To evaluate the impact of a university worksite diabetes prevention intervention on secondary outcomes regarding the change in diet quality and components of the Health Action Process Approach (HAPA) theoretical framework.	USA	HIC	HCC, NE	**NO: OOD, DB** (Alternative Healthy Eating Index (AHEI) and intake of various food groups, weight loss)	16 weeks
**2**	Socio-economic inequalities in the effectiveness of workplace health promotion programmes on body mass index: An individual participant data meta-analysis	Robroek et al. [27]	Systematic review and meta-analysis	To assess the effectiveness of workplace health promotion programmes on body mass index (BMI) across socio-economic groups and if health promotion activities could and whether study and intervention characteristics explained inequalities in effectiveness.	Netherlands	HIC	HCC, NE, AHF	**NO: OOD** (BMI)	Ranging from a few sessions (up to 5) to more than 5 sessions
**3**	Effect of a Workplace Wellness Program on Employee Health and Economic Outcomes: A Randomized Clinical Trial	Song and Baicker [18]	Randomized Clinical Trial (20 treatment worksites and 140	To evaluate the effect of a multicomponent workplace wellness program on	USA	HIC	HCC, NE	**BO** (health care spending and utilization, absenteeism, job tenure, job performance)	18 months

[1] Source for country classification by income level: World Bank Country and Lending Groups – World Bank Data Help Desk. Accessed November 30, 2022. https://datahelpdesk.worldbank.org/knowledgebase/articles/906519-world-bank-country-and-lending-groups

[2] AHF: access to healthy food; NE: nutrition education; HCC: health checks and counselling; BF: breastfeeding support.

[3] BO: business outcomes; NO: nutrition outcomes (OOD: overweight/obesity/diabetes; UA: underweight/anemia; DB: dietary behaviours; NA: nutrition knowledge/awareness); BFO: breastfeeding outcomes (EB: exclusive breastfeeding; CB: continued breastfeeding); HO: health outcomes (NCD indicators such as blood pressure and cholesterol; others).

No.	Article Title	Author(s), Year	Study Type	Primary aim(s)	Country(ies)	Income level(s)[1]	Workforce nutrition pillar(s)[2]	Outcome measures[3]	Programme duration
			control worksites)	health and economic outcomes in a middle and lower-income employee population at locations across the eastern United States.				**NO: OOD, DB** (active weight management, food choices, BMI, blood glucose)<br><br>**HO: NCDs** (regular exercise, sleep quality, alcohol consumption, smoking, cholesterol, blood pressure)	
**4**	Effectiveness of Workplace Interventions for Improving Absenteeism, Productivity, and Work Ability of Employees: A Systematic Review and Meta-Analysis of Randomized Controlled Trials	Tarro et al. [19]	Systematic review and meta-analysis of RCTs	To determine the effectiveness of workplace interventions for improving employees' work-related outcomes such as productivity, work ability, and absenteeism; and to identify the most effective methodological design for workplace interventions.	Netherlands, Denmark, USA, Japan, Germany, Finland, Australia, Brazil, Norway, Poland, Turkey, South Africa, Sweden, Switzerland	HICs and UMICs	HCC, NE	**BO** (productivity, performance, presenteeism, absenteeism, work ability)	Ranging from a one-time specific event to a maximum of 36 months
**5**	A systematic review of the cost-effectiveness of worksite physical activity and/or nutrition programs	Van Dongen et al. [28]	Systematic review	To appraise and summarize the evidence on the cost-effectiveness of worksite physical activity and/or nutrition programs.	USA, Denmark, Netherlands, Australia	HICs	AHF, HCC, NE	**BO** (absenteeism, medical costs)<br><br>**NO: OOD, DB** (weight loss, nutrient intake, waist circumference)<br><br>**HO: NCDs** (cholesterol level, cardiovascular disease risk)<br><br>**HO: others** (physical functioning, general health, vitality, mental health, impairment at work, and impairment with daily activities,	Ranging from 12 weeks to 3 years, some with follow-up ≤6 months, and some with follow-up >6 months

No.	Article Title	Author(s), Year	Study Type	Primary aim(s)	Country(ies)	Income level(s)[1]	Workforce nutrition pillar(s)[2]	Outcome measures[3]	Programme duration
								physical activity-related outcomes)	
**6**	Effectiveness of workplace weight management interventions: a systematic review	Weerasekara et al. [20]	Systematic review of randomized trials	To identify and compare the effectiveness of long-term (≥6 month) workplace weight management interventions that were tested in randomized trials, with the primary outcome being weight loss or prevention of weight gain.	USA, Denmark, UK, Japan, Australia, Finland, Israel, Netherlands, Brazil	HICs and UMC (Brazil)	HCC, NE	**NO: OOD** (weight loss, weight gain prevention, weight management, BMI, waist circumference, percentage of body fat, waist to hip ratio)	Variable, with most studies having a 6–12 months duration, and some having a 13–24-months duration
**7**	Effectiveness of workplace diabetes prevention programs: A systematic review of the evidence	Brown et al. [21]	Systematic review	To review diabetes workplace interventions and the degree to which they improve diabetes-related outcomes in employees diagnosed with or at risk for T2DM.	USA, Finland, Switzerland, Japan, Northern Ireland (including race, socio-economic, gender ethnicity considerations)	HICs	NE, HCC	**NO: OOD, DB** (A1C, body weight, BMI, blood glucose, insulin, dietary behaviours)<br><br>**HO: NCDs** (blood pressure and other vital signs, lipids, inflammatory markers)<br><br>**HO: others** (measures of depression, anxiety/stress, self-efficacy, quality of life, physical activity-related outcomes)	12-24 weeks, with outcomes commonly measured at 6 and/or 12 months. Some studies had follow-up period of up to 2-4 years post-intervention.
**8**	The effectiveness of workplace dietary modification interventions: a systematic review	Geaney et al. [25]	Systematic review of RCTs and controlled studies	To evaluate the effectiveness of workplace dietary modification interventions alone or in combination with nutrition education on employees' dietary behaviour, health status, self-efficacy, perceived health, determinants of food choice, nutrition	Brazil, USA, Netherlands, Belgium	HICs and UMC (Brazil)	AHF, NE	**BO** (absenteeism, productivity, healthcare costs, profit margins, job satisfaction)<br><br>**NO: OOD, DB, NA** (dietary behaviours, fruit and vegetables consumption, nutrient intake, determinants of food choice, nutrition knowledge, attitude to	Ranging from 3 to 24 months

No.	Article Title	Author(s), Year	Study Type	Primary aim(s)	Country(ies)	Income level(s)[1]	Workforce nutrition pillar(s)[2]	Outcome measures[3]	Programme duration
				knowledge, co-worker support, job satisfaction, economic cost and food-purchasing patterns.				food, food-purchasing patterns, BMI, waist and hip ratio measures)<br><br>**HO: NCDs** (HDL cholesterol, total cholesterol levels)<br><br>**HO: others** (perceived health, self-efficacy)	
**9**	A priority oriented nutrition education program to improve nutritional and cardiometabolic status in the workplace: a randomized field trial	Hassani et al. [11]	Randomized controlled field trial	To assess the effectiveness of a workplace nutrition education program to improve nutritional practices and cardiometabolic status in industrial personnel.	Iran	LMIC	NE	**NO: OOD, DB, NA** (nutrition knowledge, dietary behaviours, anthropometric measures, fasting blood sugar (FBS), HbA1C)<br><br>**HO: NCDs** (high-sensitivity C-reactive protein (hs-CRP), homocysteine (Hcy))	3 months
**10**	Social marketing including financial incentive programs at worksite cafeterias for preventing obesity: a systematic review	Sawada et al. [10]	Systematic review of RCTs and cluster RCTs	To assess the effectiveness of food environment interventions incorporating financial incentive or social marketing strategies at workplace cafeterias, vending machines, and kiosks toward preventing obesity and improving dietary habits.	USA, Netherlands	HICs	AHF	**NO: OOD, DB, NA** (changes in body weight, BMI, food and nutrient intakes, sales data, HbA1C)<br><br>**HO: NCDs** (blood pressure, blood lipid levels, total cholesterol, HDL and LDL cholesterol)	3 months, with follow-up at 6 and 12 months for one study
**11**	Impact of calorie labelling in worksite cafeterias: a stepped wedge randomised controlled pilot trial	Vasiljevic et al. [12]	Randomised controlled pilot trial	To identify potential barriers to the feasibility and acceptability of implementing a calorie labelling intervention; and to estimate the potential impact of calorie labelling upon	UK	HIC	NE	**NO: DB** (energy purchased from intervention items daily, and no. of food items purchased daily from intervention items and non-intervention items)	3 to 13 weeks depending on study site

No.	Article Title	Author(s), Year	Study Type	Primary aim(s)	Country(ies)	Income level(s)[1]	Workforce nutrition pillar(s)[2]	Outcome measures[3]	Programme duration
				energy purchased in worksite cafeterias.					
**12**	The effectiveness of workplace nutrition and physical activity interventions in improving productivity, work performance and workability: a systematic review	Grimani et al. [29]	Systematic review	To investigate the impact of workplace nutrition and physical activity interventions, which include components aimed at workplace's physical environment and organizational structure, on employees' productivity, work performance and workability.	USA, multiple European countries, Australia	HICs	AHF, HCC, NE	**BO** (absenteeism, presenteeism, productivity, work performance, workability)<br><br>**NO: OOD** (weight loss)<br><br>**HO: others** (sedentary behaviour, physical activity-related outcomes, musculoskeletal comfort)	Ranging from 12 weeks to a maximum of 8 years
**13**	Effectiveness of workplace interventions in Europe promoting healthy eating: a systematic review	Maes et al. [26]	Systematic review	To review and summarize the evidence of effect of intervention studies in European countries promoting a healthy diet solely and in combination with increasing physical activity at the workplace.	Multiple European countries	HICs	AHF, NE	**NO: OOD, DB** (anthropometric measures, dietary behaviours and their determinants)<br><br>**HO: others** (physical activity-related outcomes and their determinants)	Varying, some with follow-up until at least 6 months post-intervention
**14**	Effectiveness of worksite-based dietary interventions on employees' obesity: a systematic review and meta-analysis	Park and Kim [22]	Systematic review and meta-analysis of RCTs	To provide scientific evidence on the effectiveness of worksite-based dietary interventions to reduce obesity among overweight/obese employees.	USA, Denmark, UK, Australia	HICs	NE, HCC	**NO: OOD** (changes in body weight, BMI)<br><br>**HO: NCDs** (changes in total cholesterol, systolic/diastolic blood pressure)	Ranging from 3 to 12 months
**15**	Effectiveness of Interventions Targeting Health Behaviors in University and College Staff: A Systematic Review	Plotnikoff et al. [30]	Systematic review	To evaluate the literature on interventions targeting tertiary education staff within colleges and universities for improvements in health behaviours such as physical activity, dietary intake, and weight loss.	USA, Belgium, Spain, Canada, UK	HICs	AHF, NE, HCC	**NO: OOD, DB, NA** (waist circumference, weight loss, body composition, dietary behaviours, nutrition knowledge, BMI, nutrient intake)	Ranging from a 1-week to a 7-year-long intervention

No.	Article Title	Author(s), Year	Study Type	Primary aim(s)	Country(ies)	Income level(s)[1]	Workforce nutrition pillar(s)[2]	Outcome measures[3]	Programme duration
								**HO: NCDs** (blood pressure, HDL and LDL cholesterol, total cholesterol)<br><br>**HO: others** (physical activity and fitness levels, resting heart rate, seat belt use, ergonomics, smoking, alcohol consumption, perceived stress, anxiety, well-being, self-efficacy, social functioning, pain and discomfort, etc.)	
**16**	Evaluation of worksite wellness nutrition and physical activity programs and their subsequent impact on participants' body composition	Sandercock and Andrade [23]	Systematic review	To evaluate worksite wellness nutrition and physical activity programs and their subsequent impact on participants' body composition.	USA, Denmark, Germany, Japan, Netherlands	HICs	NE, HCC	**NO: OOD** (BMI, waist circumference, body fat percentage, body weight)	Varying, from one to eight years
**17**	Promoting Diet and Physical Activity in Nurses: A Systematic Review	Torquati L et al. [24]	Systematic review	To systematically review the effectiveness of intervention studies promoting diet and physical activity (PA) in nurses.	Norway, Japan, UK, Israel, USA, Switzerland, Canada	HICs	NE, HCC	**NO: OOD, DB** (BMI, fat-lean index, fat mass, waist circumference, dietary behaviours, nutrient intakes, diet self-efficacy, glucose metabolism, insulin)<br><br>**HO: NCDs** (lipid profile, blood pressure)<br><br>**HO: others** (physical activity behaviour, energy expenditure, steps, aerobic minutes, sitting time, smoking, alcohol consumption)	Short-term interventions, with the shortest being 1 and 2 days, and the longest 6 months. Some studies included follow-up at 6 and 12 months.

No.	Article Title	Author(s), Year	Study Type	Primary aim(s)	Country(ies)	Income level(s)[1]	Workforce nutrition pillar(s)[2]	Outcome measures[3]	Programme duration
**18**	The impact of worksite interventions promoting healthier food and/or physical activity habits among employees working 'around the clock' hours: a systematic review	Lassen et al. [31]	Systematic review	To assess the impact of worksite interventions to promote healthier food and/or physical activity among people who work irregular hours 'around the clock', meaning outside of ordinary daytime working hours.	Denmark, Japan, Finland, Australia, France, South Korea, USA	HICs	AHF, NE, HCC	**BO** (work performance, productivity, absenteeism, compensation claims, medical costs)<br><br>**NO: OOD, DB** (dietary behaviours, body composition, lean and fat body mass, waist circumference, weight, BMI)<br><br>**HO: NCDs** (cholesterol, cathepsin S and L)<br><br>**HO: others** (physical activity habits, VO_2 max, physical strength, well-being and quality of life, sleep circadian rhythm, psychological stress, cognitive performance, injuries at work, cumulated days with fever, cumulated time with chronic infectious diseases)	Ranging from 2 to 12 months. Only one study included a 4-year follow-up.
**19**	Systematic review on the financial return of worksite health promotion programmes aimed at improving nutrition and/or increasing physical activity.	van Dongen et al. [32]	Systematic review	To critically appraise and summarize the current evidence on the financial return of worksite health promotion programmes aimed at improving nutrition and/or increasing physical activity.	USA, Netherlands, UK	HICs	AHF, NE, HCC	**BO** (net benefits, benefit-cost ratio (BCR), return on investment (ROI) in terms of absenteeism, medical costs or presenteeism benefits)	Varying from 6 months to 5 years, with 4 studies including a follow-up post-intervention
**20**	Economic evaluation of workplace health promotion interventions focused on Lifestyle:	Vargas-Martínez et al. [34]	Systematic review and meta-analysis	To identify and evaluate randomized clinical trials focusing on economic evaluation of workplace health promotion (WHP) interventions based on	Netherlands, USA, Ireland, UK, Canada, India	HICs + India (LMIC)	AHF, NE, HCC	**BO (**absenteeism, presenteeism, intervention costs, healthcare and non-healthcare costs, health services use)	Ranging from 3 to 24 months

No.	Article Title	Author(s), Year	Study Type	Primary aim(s)	Country(ies)	Income level(s)[1]	Workforce nutrition pillar(s)[2]	Outcome measures[3]	Programme duration
	Systematic review and meta-analysis			healthy lifestyles, physical activity and nutrition.				**NO: OOD, DB, NA (**diabetes awareness score, weight loss, change in waist circumference, overweight/obesity, dietary behaviours)<br><br>**HO: NCDs (**reductions in 10-years cardiovascular disease event and mortality risk, change in blood pressure, blood glucose, lipids, cholesterol)<br><br>**HO: others** (quality of life, change in physical activity, smoking, maximal oxygen uptake, neck and upper limb symptoms)	
**21**	Strategies to improve the implementation of workplace-based policies or practices targeting tobacco, alcohol, diet, physical activity and obesity	Wolfenden et al. [33]	Systematic review and meta-analysis	1) To assess the effects of strategies for improving the implementation of workplace-based policies or practices targeting diet, physical activity, obesity, tobacco use and alcohol use. 2) To assess the impact of such strategies on employee health behaviours, including dietary intake, physical activity, weight status, and alcohol and tobacco use. 3) To evaluate their cost-effectiveness and identify any unintended adverse effects of implementation	USA, England, Brazil	HICs + Brazil (UMIC)	AHF, NE, HCC	**BO** (intervention costs)<br><br>**NO: OOD, DB** (dietary behaviours, weight status)<br><br>**HO: others** (tobacco use, physical activity level)	Ranging from 6 months to 2 years

No.	Article Title	Author(s), Year	Study Type	Primary aim(s)	Country(ies)	Income level(s)[1]	Workforce nutrition pillar(s)[2]	Outcome measures[3]	Programme duration
				strategies on workplaces or workplace staff.					
**22**	A Randomized Trial to Encourage Healthy Eating Through Workplace Delivery of Fresh Food	Feuerstein-Simon et al. [9]	Randomized controlled trial	To increase the consumption of home-cooked meals among employees at a large urban worksite through a fully subsidized Community Supported Agriculture (CSA) program.	USA	HIC (but low-wage workers)	AHF	**NO: DB (**consumption of meals prepared at home, consumption of fresh fruits and vegetables, food insecurity)	16 weeks
**23**	Effectiveness of workplace lactation interventions on breastfeeding outcomes in the United States: an updated systematic review	Kim et al. [16]	Systematic review	To evaluate the effectiveness of workplace lactation programs in the United States on breastfeeding practices.	USA	HIC	BF	**BFO (**breastfeeding initiation, exclusivity, or duration)	Policy; ongoing and continuous
**24**	Employer-based programs to support breastfeeding among working mothers: a systematic review	Dinour and Szaro [13]	Systematic review	To examine the literature regarding employer-based programs, policies, and interventions to support breastfeeding among working mothers.	10: USA, Taiwan, Indonesia, Malaysia, Thailand, Iran, Ghana, United Kingdom, Brazil, and Puerto Rico.	HICs & LMICs	BF	**BO** (job satisfaction)<br><br>**BFO** (exclusive breastfeeding and duration of breastfeeding)	Policy; ongoing and continuous
**25**	Perceptions, experiences, and outcomes of lactation support in the workplace: A systematic literature review.	Taylor et al. [15]	Systematic review	To describe employee and employer perceptions of and experiences with workplace lactation support in the United States and to identify research needed to	USA	HIC	BF	**BO** (job satisfaction)<br><br>**BFO** (exclusive breastfeeding and duration of breastfeeding, perceptions and experiences of	Policy; ongoing and continuous

No.	Article Title	Author(s), Year	Study Type	Primary aim(s)	Country(ies)	Income level(s)[1]	Workforce nutrition pillar(s)[2]	Outcome measures[3]	Programme duration
				inform workplace lactation support programs.				employees and employers with BF support programmes)	
**26**	Workplace programmes for supporting breast-feeding: a systematic review and meta-analysis.	Tang et al. [14]	Systematic review and meta-analysis	To critically review the literature regarding workplace breastfeeding interventions, and to assess their impact on breastfeeding indicators.	USA, Taiwan, Thailand, Turkey	HICs and UMICs	BF	**BFO** (exclusive breastfeeding and duration of breastfeeding)<br><br>**BO** (child illness-related maternal absenteeism)	Policy; ongoing and continuous

Data charting form 2: Key aspects of interest to this review

N.[4]	Key findings	Conclusions, lessons learned and recommendations
**1**	The experimental group lost a greater percentage of their body weight than the control group.<br>Significant difference occurred between-groups for the change in consumption of nuts/legumes and red/processed meats post-intervention and for fruits at 3-month follow-up. In particular, there was a significant increase in total Alternative Healthy Eating Index (AHEI) score post-intervention for the experimental group. The experimental group reported significantly lower intake of nuts/legumes, red/processed meats, trans-fats and sodium post-intervention than the control group and significantly greater intake of fruits at 3-month follow-up.	**Conclusions:** The worksite intervention facilitated improvement in diet quality and in planning and efficacious beliefs regarding diabetes prevention.<br><br>**Lessons learned and recommendations:** Adopting a nutrient dense diet and changing physical activity behaviors requires concerted effort and perseverance. Self-efficacy determines the amount of effort and perseverance one will invest in a behavior and personal conviction to get back on track after being derailed. Improvement in self-efficacy increase the likelihood of achieving weight loss. Greater insight into the social cognitive factors that contribute to lifestyle change will inform health behavior theory and guide the development of more effective lifestyle interventions.
**2**	A small and statistically non-significant decrease in BMI was found for the intervention groups compared with the control groups. Four out of the 16 interventions were effective compared with the control condition. No differential intervention effects were found for participants in the low socio-economic group compared with those in the intermediate or the high socio-economic group. Also, comparing the intermediate socio-economic group with the high socio-economic group, no overall interaction effect was found.	**Conclusions:** Compared with control conditions, workplace health promotion programmes overall showed a statistically non-significant decrease in BMI, which did not differ across socioeconomic positions (SEP). No evidence was found for intervention-generated SEP inequalities in BMI.<br><br>**Lessons learned and recommendations:** Interventions evaluated within randomized controlled trials, agentic interventions, those that focused on high-risk groups, included a counselling component, did not include an environmental component, consisted of more than five sessions, or were offered at the individual level did statistically significantly reduced BMI.

[4] Article numbers refer to those used in Data charting form 1.

N.[4]	Key findings	Conclusions, lessons learned and recommendations
**3**	After 18 months, the rates for 2 self-reported outcomes were higher in the intervention group than in the control group: engaging in regular exercise and actively managing weight.<br>For smoking, the unadjusted treatment group mean was -6.9 percentage points. For number of alcoholic drinks per week, the unadjusted treatment group mean was −0.6 drinks.<br>The program had no significant effects on other self-reported health outcomes and behaviors (e.g. health, sleep quality, food choices); clinical markers of health (e.g. cholesterol, blood pressure, BMI); medical and pharmaceutical spending and utilization; and employment outcomes (absenteeism, job tenure, and job performance).	**Conclusions:** Introduction of the workplace wellness program resulted in greater rates of some positive self-reported health behaviors among employees exposed compared with those who were not exposed, but there were no significant differences in clinical measures of health, health care spending and utilization, and employment outcomes (business outcomes) after 18 months.<br><br>**Lessons learned and recommendations:** Although limited by incomplete data on some outcomes, these findings may temper expectations about the financial return on investment that wellness programs can deliver in the short term.
**4**	6 out of 22 studies observed a statistically significant decrease in absenteeism (3 of which targeted employees at a high risk of sickness absences or overweight/obese). Among successful programmes, a 3-month intervention based on weight loss (comprising nutrition education and counselling) resulted in a significant decrease in the hours of absenteeism in the intervention group.<br>3 of the 11 studies reported significant results in the improvement of work ability, following physical activity interventions.<br>6 out of 22 studies observed a statistically significant improvements in productivity (productivity, performance, or presenteeism) through weight loss, stress management, cognitive-behavioral methods to improve performance, tai chi, etc. For instance, the above mentioned 3-month intervention based on weight loss significantly improved productivity as assessed by the Work Limitations Questionnaire.	**Conclusions:** The meta-analysis showed the effectiveness of workplace interventions for absenteeism, when considering only moderate quality RCTs. In contrast, only a few studies of workplace interventions for productivity and work ability were included, which was insufficient for determining the effectiveness and best design for improving these work outcomes.<br><br>**Lessons learned and recommendations:** Findings demonstrated that multi-component and counselling interventions, with virtual and individualized interventions and <10 sessions/total were the most effective methodologies to reduce absenteeism. Future high-quality RCTs that also consider health risks should be implemented to strengthen the results.
**5**	Worksite nutrition and worksite physical activity and nutrition programs were more costly and more effective in reducing body weight than usual care.<br>Most worksite nutrition and worksite physical activity and nutrition programs were more costly and more effective in reducing cholesterol level and cardiovascular disease risk, respectively.<br>One nutrition intervention was more costly and more effective in reducing daily fat/increasing daily carbohydrate intakes.<br>Another was more costly and more effective in improving general health, but less effective in reducing restraint, disinhibition (e.g. overeating due to stress) and hunger.<br>Costs for programs aimed at increasing physical activity and improving nutrition were partially offset by a reduction in absenteeism and/or medical costs.	**Conclusions:** Current evidence indicates that worksite physical activity and/or nutrition programs can result in reductions in body weight, cholesterol level, and cardiovascular disease (CVD) risk, but at a higher cost than usual care. Since it is unknown how much decision-makers are willing to pay for these health outcomes, conclusions about the cost-effectiveness of these interventions cannot be made.<br><br>**Lessons learned and recommendations:** It is established that more costly programs do not necessarily produce better health outcomes or cost-savings; therefore, cost containment strategies during the design phase of a program may be a useful strategy to optimize cost-effectiveness. There is substantial need for improvement of the methodological quality of studies evaluating the cost-effectiveness of worksite physical activity and/or nutrition programs.
**6**	Intervention effectiveness appeared unrelated to region of the world and was highest in 6–12-month trials. The results ranged widely from clinically significant 8.8-kg weight loss in one trial to less effective than the control treatment in others.<br>Most interventions reported decreases in weight and BMI outcomes compared to control arms at 6- and 12-month time points, but only 3 reported statistically significant weight loss ranging from -3.95 to −8.80 kg. One of these reported that the obtained weight loss was remarkably sustained over the follow-up period (−3.09 versus −3.95 kg).<br>The studies that lasted 13–24 months showed less mean weight loss compared to the studies in the 6–12-month group.	**Conclusions:** Study findings suggest that some workforce interventions are producing clinically meaningful weight loss with the potential for considerable sustainability over time. However, further studies are needed to replicate those results in wider sociocultural and geographical contexts.<br><br>**Lessons learned and recommendations:** The fact that the interventions causing greater weight loss are reported from US, European, and Australian workplaces suggests that workplaces in general, rather than workplaces in particular regions of the world, have great potential for obesity management once the most effective approaches have been identified and scaled.

N.[4]	Key findings	Conclusions, lessons learned and recommendations
**7**	All reviewed studies demonstrated consistent health improvements in biological measures, self-reported behavioral adherence measures, and psychosocial variables, both in comparing baseline-to-post intervention changes in single-arm studies or in comparing at least two groups at post-intervention follow-up periods.<br>Longitudinal effects in the few studies with extended outcome measurements demonstrated sustained, although ameliorated, intervention benefits.<br>The three main biological markers/outcomes consistently measured across reviewed studies were: A1C, BMI/weight loss, and blood pressure. 15 of the 20 studies that measured BMI/weight loss found statistically significant intervention effects. 6 of the 14 studies that measured blood pressure reported statistically significant improvements. 6 of the 10 studies that measured A1C reported statistically significant reductions.	**Conclusions:** The results show initial evidence that workplace interventions hold promise for preventing diabetes and/or its complications. More rigorous, creatively designed workplace studies are needed for employees at high-risk for developing diabetes.<br><br>**Lessons learned and recommendations:** For a variety of reasons, many individuals who could benefit from health guidance fail to receive important services for diabetes prevention and disease outcomes. Thus, offering such programs in the workplace where people are located is a particularly appealing strategy, because this approach circumvents many of the known barriers to accessing such programs. There is a need for employer education about the benefits of supporting such programs. Moreover, special attention must be paid to motivational strategies so employees will take full advantage of programs that are offered.
**8**	In 4 out of 6 studies, the interventions improved employees' fruit and vegetable consumption with improvements sustained over a long period of time, although small. These studies involved workplace dietary modifications and three incorporated nutrition education.<br>One study reported a statistically significant difference between the intervention and control groups for percentage of energy obtained from total fat and polyunsaturated fat.<br>One study showed a statistically significant reduction in energy intake and increase in protein and carbohydrate intakes.<br>Clinical health status outcomes were reported in one study showing a statistically significant increase in BMI and reduction of serum HDL cholesterol in the intervention group.<br>Changes in nutrition knowledge were recorded in one study and the mean score (score/10) was significantly greater in the intervention group when compared with the control group.<br>In terms of food purchasing patterns, one study found a statistically significant increase in self-purchasing of fruit and family purchasing of vegetables in the intervention groups.	**Conclusions:** Ambiguity exists including: 1) limited evidence on the long-term effects on dietary behaviour; 2) absence of information on determinants of food choice, clinical health status, and economic cost outcomes. Although available research is of limited quality, evidence suggests that workplace dietary modification interventions alone and in combination with nutrition education increase fruit and vegetable intakes.<br><br>**Lessons learned and recommendations:** Workplace dietary modification interventions should be developed with recommended guidelines, workplace characteristics, long-term follow-up and objective outcomes for diet, health and cost.
**9**	After 3 months, the intervention group showed above 50% improvement in their nutritional knowledge.<br>Body weight and BMI were lowered by 0.7 kg and 0.4 kg/m2.<br>There were no significant differences regarding the intake of sweets, soft drinks, cakes and cookies, and snacks, but the intervention group revealed significant declines in intake from the baseline.<br>Fasting blood sugar (FBS) and HbA1c levels were improved in the education group. Serum levels of high-sensitivity C-reactive protein (hs-CRP) showed no significant changes while homocysteine (Hcy) levels were reduced.	**Conclusions**: The education group significantly improved their nutritional knowledge, dietary intakes, serum FBS, HbA1C and Hcy levels, and anthropometric indices, demonstrating effectiveness of the educational program in improving CVD risk factors in employees with dyslipidemia. Workplace nutrition education programs can improve knowledge and reduce important CVD risk factors.<br><br>**Lessons learned and recommendations:** Results of the present study highlight the importance of nutrition education programs to control CVD risk factors in industrial employees. The outcomes obtained are probably due to lack of knowledge about CVD risk factors and prevention methods. Findings suggest that improved employee nutritional knowledge and dietary patterns may reduce CVD risk. Therefore, lifestyle interventions focusing on dietary pattern correction should be promoted in all worksites. Self-perception of the importance of balanced meals (i.e. nutritional knowledge) is an important factor that can influence dietary choices and intake.

N.[4]	Key findings	Conclusions, lessons learned and recommendations
**10**	One of the US trials: No significant effects on weight change, total cholesterol levels, HDL, LDL and fruit intake. No significant differences were found in energy intake or sales data between the incentive and no incentive groups.<br>One financial incentive involved a reward system instead of offering a discount for green-labeled items (fruits or vegetables, whole grains, and lean protein or low-fat dairy as the main ingredient). The percentage of green-labeled purchases was significantly greater in the incentive with feedback intervention than that in the control.	**Conclusions:** Incentive-based interventions with pricing strategies at workplaces provided no clear evidence of a significant reduction in the risk of body weight gain, but may have an influence on fruit intake. Participants were mostly obese or overweight, therefore the results may apply more to employees with a greater tendency toward obesity rather than employees in general. Neither benefits nor harms were found for other important outcomes.<br><br>**Lessons learned and recommendations:** There is a possibility that a discount-based approach at workplace cafeterias may contribute to an increased intake of fruit. Lack of evidence made it difficult to draw any conclusions. In future surveys, it will be necessary to carry out programs focusing only on financial incentive intervention versus no intervention in order to determine whether the incentive strategy has a clear impact.
**11**	There was no overall effect of calorie labelling upon energy purchased across 6 worksite cafeterias. A statistically significant reduction in total calories purchased was recorded at Site 1 (13-week intervention) on the day following the introduction of calorie labelling, but this effect diminished over time. The remaining 5 sites did not show robust changes in energy purchased when calorie labelling was introduced.	**Conclusions:** The calorie labelling intervention proved feasible and acceptable for both cafeteria operators and customers. The predicted effect of labelling to reduce energy purchased was only evident at one out of six sites studied and it was sustained over time.<br><br>**Lessons learned and recommendations:** The effects at Site 1 suggest that calorie labelling has the potential to reduce the amount of energy purchased. The lack of a significant reduction in energy purchased in five out of the six worksites may have several explanations relating to barriers to intervention implementation, the nature of the intervention, and site differences. Among the main barriers were the following: differential ability to intervene upon cafeteria products (depending on availability of energy information), chef's discretion at implementing recipes, inaccuracies in sales data, and freely available foodstuffs in the cafeterias and elsewhere across the worksites.
**12**	14 workplace nutrition and physical activity intervention studies yielded statistically significant changes on absenteeism (n = 7), work performance (n = 2), workability (n = 3), productivity (n = 1) and on both workability and productivity (n = 1).	**Conclusions:** Evidence shows that it is possible to influence work-related outcomes, especially absenteeism, positively through health promotion efforts that include components aimed at the workplace's physical work environment and organizational structure.<br><br>**Lessons learned and recommendations:** Most of the studies measured absenteeism rather than productivity, given that absenteeism data are easily and objectively assessed using workplace personnel records. Productivity on the other hand is arguably a more complex construct to measure. Due to the lack of studies that have evaluated the impact of workplace interventions on other work-related outcomes, such as workability and work performance, knowledge appears to be limited. It is therefore difficult to draw general conclusions about the effects of interventions on a particular outcome. To draw further conclusions regarding work-related outcomes, long-term follow-up using objective outcomes and/or quality assured questionnaires are required.

N.[4]	Key findings	Conclusions, lessons learned and recommendations
**13**	17 studies solely focusing on promotion of a healthy diet were reviewed, seven of which met the criteria for 'moderate' quality. These interventions showed moderate evidence for effects on body composition and dietary behaviours (e.g. fruits and vegetables intake).<br>Of the 13 studies focusing both on nutrition and physical activity, only three were rated as having 'moderate' quality, providing inconclusive evidence for effects.	**Conclusions:** Limited to moderate evidence was found for positive effects of nutrition interventions implemented at the workplace. None of the types of interventions reviewed consistently produced significant effects on anthropometric measures. Combined nutrition and physical activity interventions showed less positive results.<br><br>**Lessons learned and recommendations:** Effects of workplace health promotion interventions may be improved if stronger adherence to established quality criteria for such interventions is realized.
**14**	Results showed that weight decreased with weighted mean difference (WMD) of -4.37 and BMI also decreased with WMD of -1.26, but the effectiveness was statistically significant only in short-term programs (< 6 months). In studies that implemented long-term programs (> 6 months), there was a larger decrease in weight (WMD -4.56 kg) and BMI (WMD -1.68 kg/m2) than in the short-term programs, but with no statistical significance.<br>Total cholesterol decreased, demonstrating significant effectiveness.<br>Both systolic and diastolic blood pressure decreased, demonstrating effectiveness, but with no statistical significance.	**Conclusions:** The worksite-based dietary interventions for overweight/obese employees showed modest short-term effects. These interventions can be considered successful because weight loss was below approximately 5-10 kg of the initial body weight, which is the threshold for the management of obesity recommended by the Scottish Intercollegiate Guideline Network (SIGN).<br><br>**Lessons learned and recommendations:** The difference in study outcomes according to program duration implies that worksite-based dietary interventions targeting overweight or obese employees are more effective in short-term programs (< 6 months), possibly because participants can better focus their attention on and are more aware of their diet. However, long-term weight loss maintenance does not seem impossible. Evidence showed that some participants succeeded in long-term weight loss, through strategies targeting diet, physical activity and other behavioral aspects. Habit formation is influenced by the surrounding environment; therefore, the generation of a healthy food environment at the workplace is likely to result in a more positive contribution to maintain the effect of dietary intervention programs for employees with obesity.
**15**	Weight loss, physical activity and fitness, and/or nutrition were the focus in 9 of the 17 included studies. The review yielded overall positive results with 13 studies reporting significant health-related improvements.<br>Of the three studies that assessed nutrition-related behaviours, all reported significant improvements.<br>Five studies reported a significant effect of the intervention on body weight and/or body composition.<br>The five studies aimed at reducing chronic disease risk, also recorded significant findings.<br>In particular, statistically significant differences in HDL cholesterol, total cholesterol to HDL ratio, and diastolic blood pressure were found between groups.	**Conclusions:** Most studies resulted in at least one significant improvement in a nutrition or health outcome, and a number of studies had multiple positive effects.<br><br>**Lessons learned and recommendations:** There is scope to enhance cross-disciplinary collaboration in the development and implementation of a "Healthy University" settings-based approach to health promotion in tertiary education workplaces. Universities or colleges could serve as a research platform to evaluate such intervention strategies.
**16**	17 out of 23 studies resulted in a positive change in body composition (decreased BMI, waist circumference, and body fat percentage), while 6 studies did not show any changes.<br>Programs that had professionals frequently interact with participants, regardless if the interactions were daily, weekly, or monthly, always led to a change in body composition.<br>Additionally, programs that incorporated a motivation theory component and provided content relevant to participants' needs resulted in a change in body composition.	**Conclusions:** Evidence showed that worksite wellness programs that are designed using a motivational theory and whose content is developed based on participants' needs, and that include frequent interactions between professionals and participants may result in a positive change in body composition. Participants who were able to interact with others on a consistent basis (e.g. interactive websites, group discussions, or one-on-one health coaching) were more likely to change their body composition.

N.[4]	Key findings	Conclusions, lessons learned and recommendations
		**Lessons learned and recommendations:** While self-motivation theories and intensive educational sessions have been identified as effective components, there are more elements of programs to explore such as health assessments, health marketing, and health campaigns. This review contributes to identifying the most effective and beneficial components of worksite programs, so that future worksite wellness programs could be modeled using only favorable elements to warrant positive results.
**17**	Dietary outcomes were generally positive, but were only measured in 4 studies with some limitations in the assessment methods. In particular, among the 6 interventions that included a diet and nutrition component, 2 did not assess any dietary outcomes, whereas the others presented heterogeneous outcomes (e.g. fruit and vegetable intake, diet self-efficacy, diet behavior based on a general lifestyle tool score).<br>6 studies assessed different body composition parameters as secondary outcomes, but only two of them found significant changes. Overall, there were modest improvements on participants' BMI and body composition.	**Conclusions:** Outcomes of interventions to change nurses' physical activity and diet behavior are promising, but inconsistent. Although there was a modest increase in some measures of physical activity and a positive effect on participants' BMI and body composition, results should be interpreted with caution.<br><br>**Lessons learned and recommendations:** Additional and higher quality interventions that include appropriate theoretical frameworks and objective and validated outcome measures and appropriate process evaluation are required. Moreover, interventions should be feasible, tailored to participants' needs and responsive to their feedback.
**18**	All 3 studies (out of 7) evaluating dietary habits found some significant improvement, for example: an increased intake of fruit and vegetables, a decreased intake of sweetened beverages and an increased intake of water. In one of the 3 studies, mediating factors for the improvement in fruit and vegetable intake included an increased knowledge of their health benefits and an improved level of social support (dietary norms) experienced from co-workers. The only study which included a 4-year follow-up period concluded that 1-year effects of the programme did not remain over time compared to the control group, but the long-term pattern of behaviours in both groups suggested that the worksites, as a whole, had continued to improve in outcome measures for several years following the programme.<br>All 3 studies which evaluated blood measures (cholesterol and the biomarker cathepsin) found significant positive effects: decreased level of LDL-cholesterol, increased level of HDL cholesterol and a decrease in cathepsin S and L.<br>Body composition was assessed in 6 out of 7 studies, but only 2 of them found a significant positive effect of the intervention.<br>Weight and BMI were measured in 6 out of 7 studies. Results varied from significant difference in weight loss between intervention and control groups, to no significant weight changes, to less weight gain in the intervention group than the control group.<br>Impact on work performance was assessed in 3 studies, all with positive outcomes. One study assessed work place productivity, injuries at work and absenteeism and found a positive effect on all these outcomes. Another one found a reduction in compensation claims and medical costs. A third study reported a positive significant effect on workers' cumulated days with fever, but not on cumulated time with chronic infectious diseases.	**Conclusions:** The studies showed small-to-moderate effect sizes on several measures, including dietary and/or physical activity measures, suggesting acceptable effectiveness for interventions involving community-level behaviour change.<br><br>**Lessons learned and recommendations**: There is a need to further develop and implement well-designed health promotion interventions with comparable outcome measures and effect size reports. A mixture of health promotion strategies is recommended for future practice in this target population, including individually tailored programmes, improving the food and physical activity environment and using broader lifestyle approaches including the use of participatory and empowerment strategies.
**19**	The financial return was positive in 14 out of 21 interventions.<br>The net benefits (NB) ranged from -$451 to $2,757 (median: $91, n = 21), indicating the amount of money gained after costs were recovered.	**Conclusions:** On average, the financial return in terms of absenteeism benefits, medical benefits or both were positive during the first years after implementation.

N.[4]	Key findings	Conclusions, lessons learned and recommendations
	The benefit-cost ration (BCR) ranged from -0.76 to 18.84 (median: 1.42, mean: 3.76, SD: 5.36), indicating the amount of money returned per dollar invested. The ROI ranged from -176% to 1,784% (median: 42%, mean: 276%, SD: 536%), indicating the percentage of profit per dollar invested.	However, while average financial return estimates in terms of absenteeism benefits (reduced absenteeism costs), medical benefits (reduced medical costs) or both were positive in non-randomized studies (NRSs), but negative in randomized controlled trials (RCTs). Since these programmes are associated with additional types of benefits, conclusions about their overall profitability cannot be made.<br><br>**Lessons learned and recommendations**: Findings of the present review indicate that financial return estimates derived from NRSs should be interpreted with caution. RCTs with a low risk of bias indicate that workplace programmes aimed at improving nutrition and/or increasing physical activity do not pay for themselves in terms of reduced absenteeism costs, medical costs or both during the first years after implementation. This is in contrast with the conclusions of previous reviews. An explanation for this discrepancy may be that the previous reviews were mainly based on NRSs, which might have confounded their results.
**20**	Several lifestyle interventions assessing triglycerides, LDL and HDL cholesterol, and total cholesterol found statistically significant differences between intervention and control group, resulting in positive outcomes of the intervention applied. Two studies also showed the cost-effectiveness of interventions in terms of cost savings to society and employers associated with significant reductions in CVD risk.<br>4 lifestyle intervention studies found statistically significant weight losses. 2 of these studies also reported statistically significant reductions of the waist circumference in the intervention group compared with the control group. In addition, one of these studies reported a statistically significant difference in BMI between intervention and control groups. The last study also resulted in a reduction of absenteeism generating high net benefits (€53.56 per employee). Another study found statistically significant differences between intervention and control group related to presenteeism (52h).<br>5 interventions were included in the meta-analysis, which showed a general decrease of −0.61 Kg in weight, but not in waist circumference.	**Conclusions:** Study findings demonstrate the effectiveness of the workplace health promotion interventions, and in some cases (about one-third), the efficiency of these interventions for both employers and society. The majority of interventions were either partially or fully effective in achieving their objectives (decreased LDL cholesterol, reduced CVD risk, increased physical activity, weight loss, decreased absenteeism etc.)<br><br>**Lessons learned and recommendations**: Additional research in this area is necessary as well as the assessment of the cost-effectiveness of such interventions. The majority of successful interventions comprised counselling/coaching through different channels, and system-level modifications at the workplace (e.g. menu modification, price discounts on fruit, strategic positioning of healthier alternatives and portion size control).
**21**	3 trials examining the impact of implementation strategies on employee health behaviours, reported mixed effects for diet and weight status and no effect for physical activity or tobacco use. One trial found an increase in absolute workplace costs for health promotion in the implementation group.	**Conclusions:** Available evidence regarding the effectiveness of implementation strategies for health-promoting policies and practices in the workplace setting is sparse and inconsistent. Low certainty evidence suggests that such strategies may make little or no difference on measures of implementation or employee health behaviour outcomes. It is also unclear if such strategies are cost-effective. The meta-analysis found no difference in standardised effects, suggesting no benefit of implementation support in improving policy or practice implementation, relative to control.<br><br>**Lessons learned and recommendations**: The limited number of trials identified suggests implementation research in the workplace setting is in its infancy. Further research is needed to guide evidence translation in this setting.
**22**	8 biweekly full subsidized fresh food deliveries through a community supported agriculture (CSA) program plus cooking education and support. Control participants received usual employee benefits.Compared to the control group, intervention participants consumed 29%	**Conclusions**: The study demonstrated the feasibility and potential positive effects of a subsidized workplace CSA program, augmented with cooking education and support. Observed benefits included increased consumption of home-cooked meals

N.[4]	Key findings	Conclusions, lessons learned and recommendations
	more home-cooked meals per week (P < .01). Fruit and vegetable consumption also increased among intervention participants. The odds of at least twice-daily fruit consumption were 3.8 times higher among intervention participants than among controls, and the odds of at least twice-daily vegetable consumption were 6.2 times higher among intervention participants than among controls. Compared to control participants, intervention participants experienced a statistically significant 89% reduction in the odds of reporting food insecurity at follow-up, when controlling for baseline food insecurity. Participants reported perceived intervention benefits, including the opportunity to experiment with new, healthful foods without financial risk, as well as the social value of sharing recipes, food, and related conversation with colleagues.	and of fruits and vegetables. In addition, the study showed that CSA participation reduced the odds of experiencing food insecurity.<br><br>**Lessons learned and recommendations:** To assess longer-term sustainability of these changes and impact on health outcomes, as well as barriers and facilitators to implementing similar programs at other worksites, further research is needed. Such information would be useful to employers considering implementing similar worksite wellness efforts.
**23**	Common services provided were breast pumps, social support, lactation rooms, and breastfeeding classes. Breastfeeding initiation was very high, ranging from 87% to 98%. Several factors were significantly associated with duration of exclusive breastfeeding: (a) receiving a breast pump for one year (8.3 versus 4.7 months), (b) return-to-work consultations (40% versus 17% at 6 months), and (c) telephone support (42% versus 15% at 6 months). Each additional service (except prenatal education) dose-dependently increased exclusively breastfeeding at 6 months. Sociodemographic information including older maternal age, working part-time, longer maternity leave, and white ethnicity were associated with longer breastfeeding duration.	**Conclusions:** Comprehensive lactation programs have been shown to increase breastfeeding duration among mothers who were planning to breastfeed before maternity leave in middle- to high-income families.<br><br>**Lessons learned and recommendations:** Workplace lactation interventions increased breastfeeding initiation, duration, and exclusive breastfeeding, with greater changes observed with more available services. More evidence is needed on the impact of workplace support in low-income populations, and the cost-effectiveness of these programs in reducing health care costs.
**24**	Providing a lactation space was the most common employer-based support accommodation studied, followed by breastfeeding breaks and comprehensive lactation support programs. The majority of studies analyzing these three support types found at least one positive breastfeeding and/or nonbreastfeeding outcome.<br>Among the studies analyzing breastfeeding duration, all showed that women with access to a comprehensive lactation support program (3 or more support offerings) at work either had significantly lower rates of breastfeeding discontinuation upon returning to work,32 longer duration of breastfeeding,32 higher rates of any breastfeeding at 6 months27–29,37 and 12 months,29 longer duration of exclusive breastfeeding,10,32 and higher rates of exclusive breastfeeding at 6 months.	**Conclusions:** This review suggests that maintaining breastfeeding while working is not only possible but also more likely when employers provide the supports that women need to do so. Although some employers may have more extensive breastfeeding support policies and practices than others, all employers can implement a breastfeeding support program that fits their company's budget and resources.<br><br>**Lessons learned and recommendations**: Given the current lack of controlled studies, all studies included in the review utilized an observational research design. More rigorous controlled studies will need to be conducted to confirm these findings, using a uniform definition of breastfeeding support programmes.
**25**	27 studies included in the review all from USA, Analyses of associations between lactation support at work and employee breastfeeding outcomes (*n* = 14, 52%), and employee perceptions of and experiences with lactation support at work (*n* = 14, 52%) were most common, followed by employer reports of lactation support (*n* = 3, 11%) and associations between lactation support at work and job satisfaction (*n* = 3, 11%).. The use of cross-sectional designs, unvalidated instruments, and limited representation from women with low incomes and minorities were common study limitations.	**Conclusions:** Results indicated that workplace lactation support varied by employer, and that employee perceptions of and experiences with workplace lactation support varied by demographic and employment characteristics.<br><br>**Lessons learned and recommendations:** More research is needed to learn about experiences of employers and low-income and minority women with workplace lactation support and associations with business-relevant outcomes.

N.[4]	Key findings	Conclusions, lessons learned and recommendations
**26**	The pooled odds ratio for exclusive breastfeeding at 3 or 6 months for participants vs. non-participants of three non-randomised controlled studies was 3.21 (95% CI 1.70, 6.06, I2=22%). In all 5 studies looking at EBF, participants in workplace programs (after improving the law regulating maternity leave in one study(42)) were more likely to practice exclusive breastfeeding.<br>Despite high heterogeneity, other pooled outcomes were consistently in a positive direction with acceptable confidence intervals. Pooled mean duration of breastfeeding for five single-armed studies was 9.16 months (95% CI 8.25, 10.07). Pooled proportion of breastfeeding at 6 months for six single-armed studies was 0.76 (95% CI 0.66, 0.84) and breastfeeding at 12 months for three single-armed studies was 0.41 (95% CI 0.22, 0.62).<br>Results on infant-illness related maternal absenteeism results suggested that employers also benefited from offering a workplace breastfeeding support program to employees.	**Conclusions:** Workplace programs may be effective in promoting breastfeeding among employed mothers and partners of employed fathers. Business outcomes are also likely to be positive.<br><br>**Lessons learned and recommendations:** No randomised controlled trials were identified, and better-quality research on workplace interventions to improve breastfeeding is needed.
